# Squamous Cell Carcinoma In Situ of the Breast With the Configuration of an Intraductal Papilloma

**DOI:** 10.7759/cureus.49382

**Published:** 2023-11-25

**Authors:** Maria R Mayorga, Gloria Hutchinson

**Affiliations:** 1 Pathology, University of Tennessee Graduate School of Medicine, Knoxville, USA; 2 Pathology and Laboratory Medicine, Brookwood Baptist Health, Birmingham, USA

**Keywords:** in situ carcinoma, scc, malignant transformation, squamous metaplasia, intraductal papilloma, breast cancer

## Abstract

A 64-year-old woman presented to our institution with a palpable and painful left breast mass. She denied any other breast symptoms. Subsequent imaging classified it as a US Breast Imaging-Reporting and Data System (BI-RADS) 4A lesion. A core needle biopsy was performed showing atypical proliferating fragments of squamous epithelium suspicious for malignant neoplasm. An excisional biopsy was recommended. Gross examination showed a well-circumscribed pink soft mass measuring 2.0 x 1.4 x 1.3 cm. The entire lesion was submitted for histologic evaluation, demonstrating a neoplasm with branching stroma and exuberant squamous differentiation. The lesion exhibited obvious cytologic features of malignancy like mitotic figures, prominent nucleoli, irregular nuclei, and multinucleation. Collagen IV stain ruled out invasion. The lesion was finally classified as squamous cell carcinoma (SCC) in situ with the configuration of an intraductal papilloma. The possibility of metastatic disease was suggested. A PET scan was negative, and no other foci of disease were found in the remainder of the specimen. The mass was also independent of nipple and skin. Based on the architectural features, we believe that this is a case of an intraductal papilloma that underwent complete squamous metaplasia with no residual adenomyoepithelial components and transformation into an SCC in situ demonstrated by stains. Papillomas can undergo reactive metaplastic changes, usually benign and in small foci. This is the first reported case of exuberant squamous epithelium that transformed into carcinoma in situ with papillary architecture in the breast.

## Introduction

Intraductal papillomas (IDPs) are benign lesions that grow into a breast duct and can present at any age. They are more frequently found in the subareolar region often appearing as a palpable mass with associated nipple discharge [1]. Histologically, these lesions consist of a branching stroma with columnar and myoepithelial cells. IDPs have been known to undergo metaplastic changes (apocrine, squamous, sebaceous) as a reactive process [1]. Squamous metaplasia is usually benign and presents as a small focus in all the cases we found in the literature. This is the case of a patient with an IDP that underwent complete squamous metaplasia and malignant transformation.

## Case presentation

**Figure 1:**
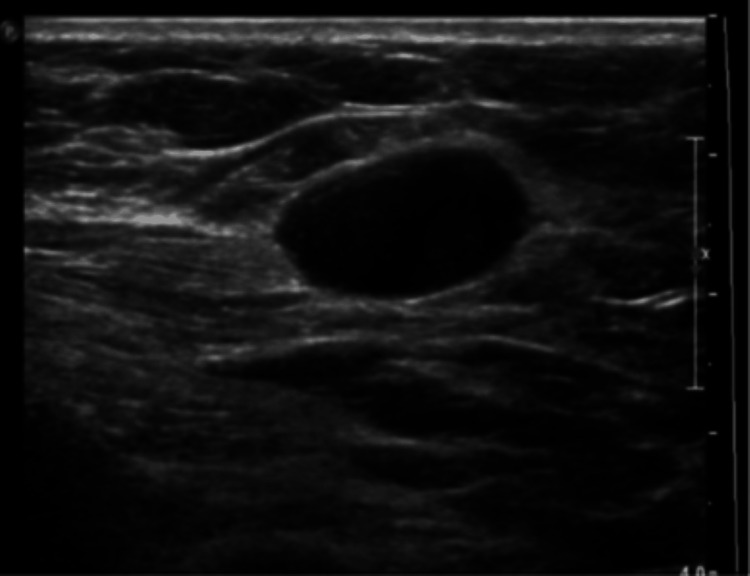
Breast ultrasound BI-RADS: Breast Imaging-Reporting and Data System US BI-RADS 4A lesion was identified.

A 64-year-old woman with a past medical history of hypertension and no relevant past surgical or family history presented to our institution with a palpable and painful left breast mass. A previous mammogram performed at an outside facility showed a Breast Imaging-Reporting and Data System (BI-RADS) 0 mass in the upper outer quadrant of the left breast. Subsequent imaging classified it as a US BI-RADS 4A lesion (Figure 1). She denied any other breast symptoms. On physical exam, her breast was normal appearing with a left upper outer quadrant palpable tender mass.

**Figure 2:**
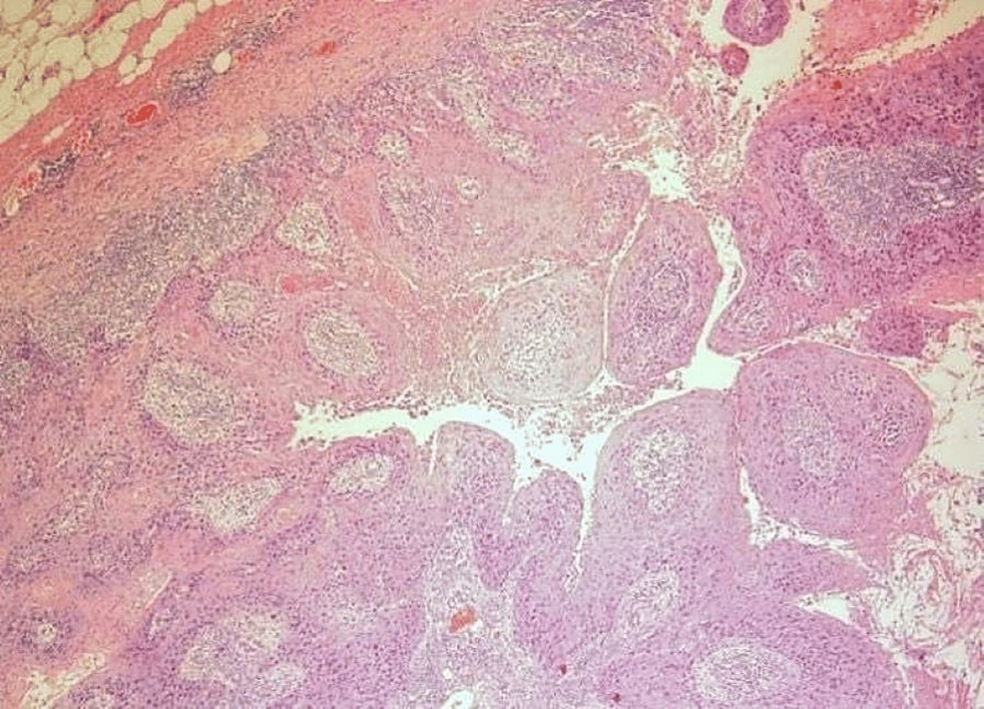
H&E 40x Edge of lesion showing circumscribed intraductal proliferation comprised of arborizing fibrovascular cores lined by squamous epithelium.

**Figure 3:**
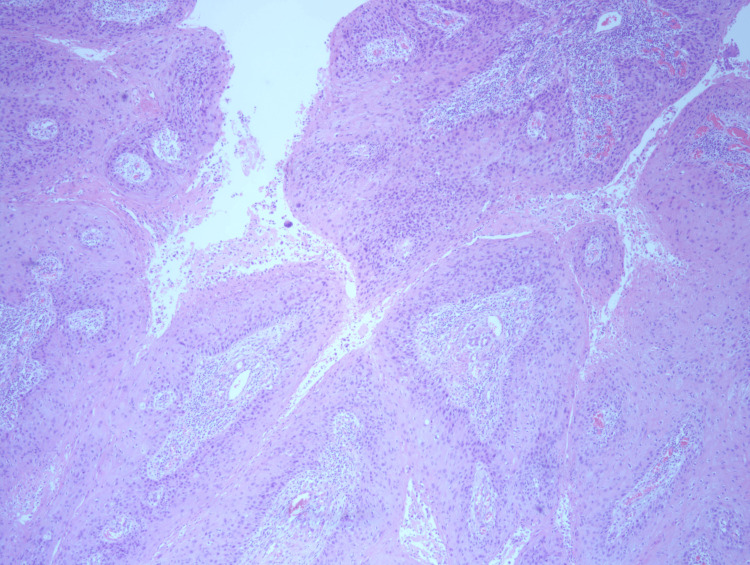
H&E 40x Florid squamous metaplasia with the architectural configuration of a papilloma.

**Figure 4:**
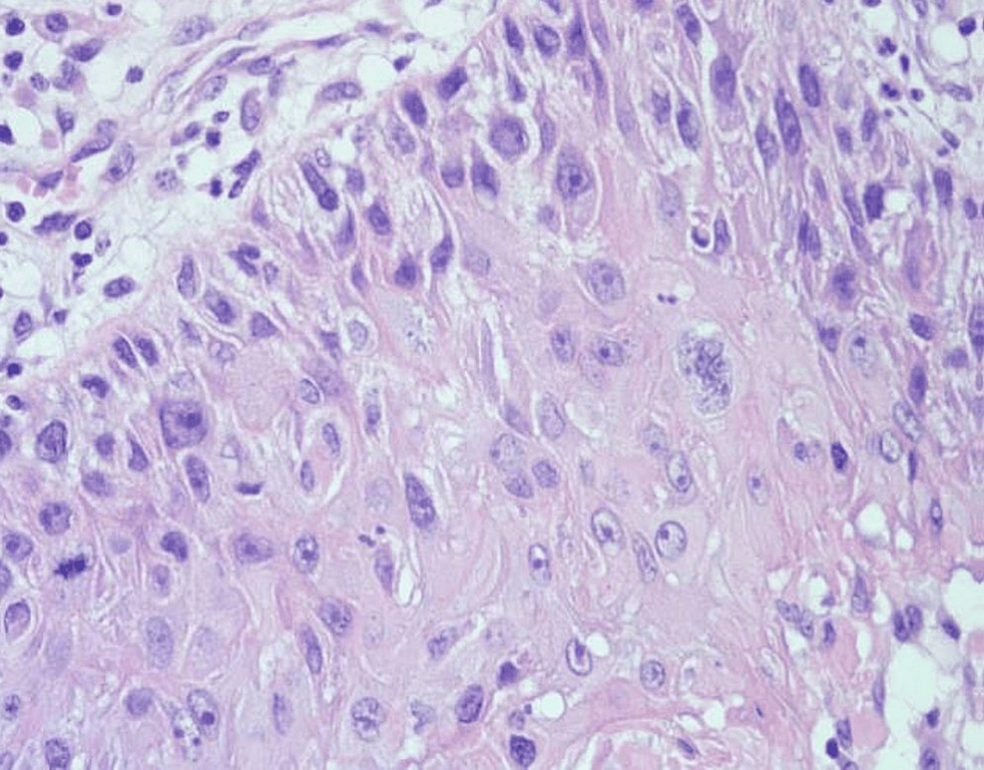
H&E 400x Squamous epithelium demonstrating cytologic evidence of malignancy. Mitotic figures, multinucleation, pleomorphism, and prominent nucleoli are visible.

**Figure 5:**
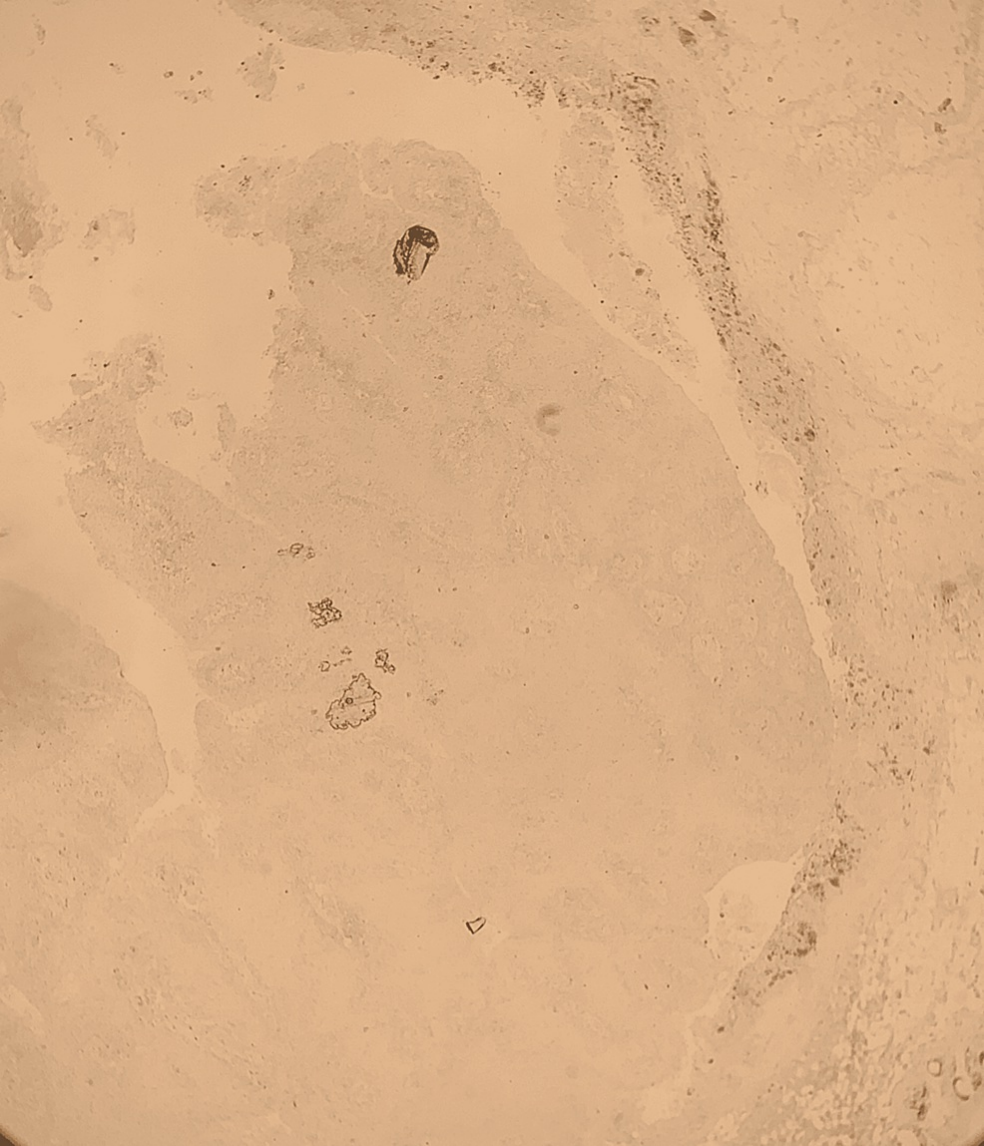
Collagen IV The stain shows the in situ nature of the lesion.

A core needle biopsy was performed showing atypical proliferating fragments of squamous epithelium with necrotic and keratotic debris. The fragments were atypical and suspicious, though not alone diagnostic for malignant neoplasm. The patient underwent an excisional biopsy. Gross examination showed a well-circumscribed pink soft mass measuring 2.0 x 1.4 x 1.3 cm. The entire lesion was submitted for histologic evaluation demonstrating a papillary neoplasm with exuberant squamous differentiation (Figure 2, Figure 3, and Figure 4). All margins were negative. Collagen IV stain ruled out invasion (Figure 5). The cytologic features were compatible with malignancy. The lesion was finally classified essentially as squamous cell carcinoma (SCC) in situ with the configuration of an IDP. Due to the rarity of primary SCC of the breast, a PET scan was performed to rule out an alternative site. The scan was negative. Follow-up with radiation oncology was recommended, and no further treatment was necessary. The patient was lost to transfer of care.

## Discussion

Reactive changes in an IDP can lead to squamous metaplasia which is usually benign. It can occur de novo, after infarction, or after a needling procedure [1]. Most cases found in the literature mention metaplastic changes making up a small focus of the lesion. Ginter et al. [2] described a rare case of an IDP with exuberant squamous metaplasia; in this case, the metaplastic epithelium constituted 30% of the lesion with the remainder demonstrating the typical adenomyoepithelial elements. The squamous epithelium was bland and non-infiltrative which ruled out the possibility of carcinoma. Meanwhile, the mass found in our patient demonstrated the architectural preservation of an IDP with the entirety of the lesion submitted for histologic evaluation showing 100% squamous differentiation. No residual adenomyoepithelial components were identified within the lesion.

Flint and Oberman [3] reported eight cases of infarcted IDPs with squamous metaplasia present in three; in those cases, fibrous tissue contributed to the distortion and compression of isolated foci of metaplastic cells giving the appearance of an infiltrative carcinoma. The authors concluded that in cases of squamous metaplasia and infarction, the presence of neoplasm elsewhere in the biopsy and cytologic features should be used to determine the presence of malignancy. In the case of our patient, the lesion exhibited clear cytologic features of malignancy like mitotic figures, prominent nucleoli, irregular nuclei, and multinucleation. Considering that primary SCC of the breast is exceedingly rare, making up less than 0.1% of breast cancers [4], and most of them are a result of metastatic disease or direct invasion from the overlying skin and nipple, the possibility of metastasis was suggested. However, no other foci of disease were found in the remainder of the excisional biopsy specimen, and the mass was independent of nipple and skin. The PET scan also ruled out the presence of another primary, and a collagen IV stain confirmed this to be an in situ process.

## Conclusions

Based on the architectural features and presence of exuberant squamous epithelium with no remnants of adenomyoepithelial cells, no evidence of invasion, or an alternative primary, this is most likely an intraductal IDP that underwent complete metaplastic changes with subsequent malignant transformation into an SCC in situ. The presence of necrotic material in the initial core needle biopsy also suggests that this was a de novo process and not a result of a previous procedure.
